# 2582. Real-World Analysis of the Outcomes of Patients Treated with Ceftazidime-Avibactam

**DOI:** 10.1093/ofid/ofad500.2197

**Published:** 2023-11-27

**Authors:** Rayyan Wazzi-Mkahal, Reem Mansour, Mahdi Hamade, Racha Ghoussaini, Adeeb Tashman, George Doumat, Antoine Abou-Fayad, Ghassan Matar, Souha S Kanj, Zeina Kanafani

**Affiliations:** American University of Beirut, Beirut, Beyrouth, Lebanon; American University of Beirut, Beirut, Beyrouth, Lebanon; American University of Beirut, Beirut, Beyrouth, Lebanon; American University of Beirut, Beirut, Beyrouth, Lebanon; American University of Beirut, Beirut, Beyrouth, Lebanon; American University of Beirut, Beirut, Beyrouth, Lebanon; American University of Beirut, Beirut, Beyrouth, Lebanon; American University of Beirut, Beirut, Beyrouth, Lebanon; American University of Beirut Medical Center, Beirut, Beyrouth, Lebanon; American University of Beirut Medical Center, Beirut, Beyrouth, Lebanon

## Abstract

**Background:**

Ceftazidime-avibactam (CAZAVI) is a novel β-lactam/β-lactamase inhibitor combination with activity against *Pseudomonas aeruginosa* and some carbapenem-resistant Enterobacterales. CAZAVI has been particularly useful as a carbapenem-sparing strategy to mitigate the selection pressure observed with carbapenems. We aim to perform a real-world analysis of patients treated with CAZAVI for various clinical indications at a tertiary care center in Lebanon.

**Methods:**

This is a retrospective study conducted at the American University of Beirut Medical Center from 2019-21. We excluded patients who were less than 18 years old, received CAZAVI for less than 48 hours, had severe COVID-19 infection, received more than three courses of CAZAVI, and patients on palliative care before CAZAVI treatment. Data were collected from electronic medical records. The primary endpoint was unfavorable outcome defined as death or incomplete clinical response.

**Results:**

A total of 301 patients were included contributing 373 infection episodes. The median age was 66 years, with 48% of patients being male, and 55% having a high Charlson Comorbidity Index (CCI) of ≥ 5. A large proportion of patients had been admitted to the intensive care unit and had received antibiotics for more than 48 hours during the preceding 30 days. The majority of infection episodes were hospital-acquired (66%). The most common indication for CAZAVI was respiratory infection (44%) followed by bacteremia (9%). Clinical cultures were positive in 44% of episodes distributed as Enterobacterales 64%, *P. aeruginosa* 10%, and polymicrobial 26%. Clinical success was 66% and microbiological success was 52%. Mortality was 32%, 65% of which was directly attributable to the infection. The primary endpoint was achieved in 38% of episodes. Multivariate analysis of independent predictors of unfavorable outcome is shown in the Table.

Independent Predictors of Unfavorable Outcomes in Patients Receiving CAZAVI

**Figure:**
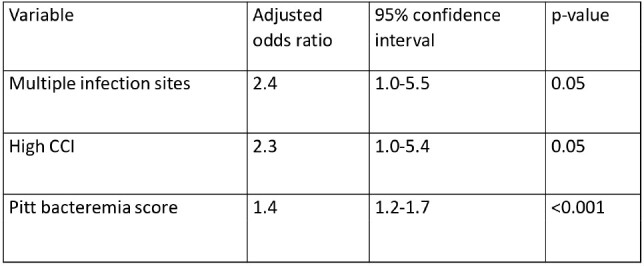

**Conclusion:**

Results from this highly morbid patient population suggest a good overall response to CAZAVI therapy. The presence of multiple infection sites is an important predictor of unfavorable outcome.

**Disclosures:**

**Souha S Kanj, MD**, Gilead: Advisor/Consultant|Menarini: Advisor/Consultant|MSD: Advisor/Consultant|Pfizer: Advisor/Consultant

